# Effects of Mesenchymal Stromal Cell-Derived Extracellular Vesicles on Tumor Growth

**DOI:** 10.3389/fimmu.2014.00382

**Published:** 2014-08-11

**Authors:** Stefania Bruno, Federica Collino, Alessandra Iavello, Giovanni Camussi

**Affiliations:** ^1^Department of Molecular Biotechnology and Health Sciences, University of Torino, Torino, Italy; ^2^Department of Medical Sciences, University of Torino, Torino, Italy

**Keywords:** mesenchymal stem cells, extracellular vesicles, anti-tumor effect, pro-tumorigenic activity, *in vivo* tumor models

## Abstract

Extracellular vesicles (EVs) are membrane vesicles, which are secreted by a variety of cells that have a relevant role in intercellular communication. EVs derived from various cell types exert different effects on target cells. Mesenchymal stromal cells (MSCs) are stem cells that are ubiquitously present in different tissues of the human body, and MSC-derived EVs take part in a wide range of biological processes. Of particular relevance is the effect of MSCs on tumor growth and progression. MSCs have opposing effects on tumor growth, being able either to favor angiogenesis and tumor initiation, or to inhibit progression of established tumors, according to the conditions. Different studies have reported that EVs from MSCs may exert either an anti- or a pro-tumor growth effect depending on tumor type and stage of development. In this review, we will discuss the data presented in the literature on EV-mediated interactions between MSCs and tumors.

## Introduction

Mesenchymal stromal cells (MSCs) are multipotent cells that reside in various tissues, and possess the capacity to differentiate into different mesodermal lineages (1–8). MSCs can be recruited to the site of inflammation and tissue injury/repair, as well as within the tumor environment (9–12). In this context, several studies have shown that MSCs may support tumor growth *in vivo* (13–17), whereas others have reported an anti-tumorigenic effect for these cells (18–23). MSCs isolated from different tissues, such as human adipose tissue (24), breast (25), and palatine tonsils (26), have been shown to have the capacity to interfere with cancer cell proliferation, blocking tumor cell cycle in G0/G1 phases.

The different effects of MSCs on tumor growth depend on the tumor models, but also on the dose and time of administration of cell treatments (12). In particular, MSCs co-injected with tumor cells have been shown to support angiogenesis, thus facilitating tumor growth (13–17, 27). Conversely, intravenous or intra-tumor injection of MSCs in established tumors led to inhibition of tumor growth (18–21, 24). The exact mechanisms of these opposite effects remain unclear.

*In vitro* experiments have shown that cell contact between MSCs and tumor cells is not required for MSC biological activity, as the anti-proliferative effect was also observed with MSC-conditioned medium (22, 28). This observation has led to the suggestion that paracrine/soluble factors are involved instead.

Extracellular vesicles (EVs) (exosomes and shedding microvesicles) are nano-particles secreted by various cell types, which contain protein, lipids, and genetic material, such as mRNA and miRNA. Transfer of this biological material to adjacent or distant cells may facilitate communication between different cell types. Secreted EVs express molecules that reflect the cells of origin and, in the field of regenerative medicine, EVs derived from MSCs (MSC-EVs) have been shown to be able to mimic the therapeutic effects of the MSCs in kidney, cardiac, and brain injuries (29, 30). EVs released from MSCs could also be involved in the effects of MSCs on tumor growth and behavior. Several studies describing the influence of MSC-EVs on tumor growth have been reported. Similar to the case of MSCs, the released EVs can also have opposite effects on tumor growth, depending on the tumor type and the experimental animal models.

## State of the Art on MSC-EV Content

Mesenchymal stromal cell-EVs express surface molecules that are characteristic of the cells origin, such as CD29, CD73, CD44, and CD105 (31). Moreover, MSC-EVs contain cytoplasmatic proteins associated with intracellular vesicle biogenesis and trafficking (RAB protein family), and proteins associated with MSC self-renewal and differentiation (TGF-β, MAPK, PPAR, etc.) (32).

Mesenchymal stromal cell-EVs also contain nucleic acids (mRNA and non-coding RNA). The mRNAs present in EVs are representative of the multiple differentiation and functional properties of MSCs, including transcripts related to several different cell functions (e.g., the control of transcription, cell proliferation, and immune regulation) (33). EVs from MSCs also contain mRNA for receptors of specific growth factors, such as mRNA for the insulin growth factor 1 (IGF-1) receptor (34). MSC-EVs are able to transfer the IGF-1 receptor mRNA to target renal tubular cells in an *in vitro* model of renal toxic injury, inducing proliferation of proximal tubular cells (34).

EVs released by MSCs, also contain specific non-coding RNA, such as miRNAs. miRNAs are small non-coding RNAs that regulate gene expression post-transcriptionally by targeting specific mRNAs. EVs from different cell types have been shown to contain selected patterns of miRNAs (35, 36), which can be subsequently transferred to target cells (36, 37). The EV-shuttled miRNAs were functionally active, evident from their ability to down-regulate proteins targeted by selected transferred miRNAs (36–39).

Gene ontology analysis of the molecules targeted by the highly expressed miRNAs in MSC-derived EVs revealed genes involved in multi-organ development, cell survival, and differentiation (36).

## Anti-Tumor Effect of MSC-EVs

It has been demonstrated that MSC-EVs inhibited the proliferation of HepG2 hepatoma, Kaposi’s sarcoma (KS), and Skov-3 ovarian cancer cell lines, *in vitro* (40). Specifically, MSC-EVs increased the percentage of tumor cells in G0/G1 phase, indicating a block in cell cycle progression. Moreover, in hepatoma and KS cancer cell lines, MSC-EV treatment induced apoptosis, as demonstrated by cytofluorimetric analyses (sub-G1 peak in cell cycle studies and activation of Caspase 8 and/or 9) and by Tunel assay. By contrast, in Skov-3 cells, EVs induced cell death by necrosis. Gene array profiles showed that, after 24 h of *in vitro* stimulation with MSC-EVs, different genes were modulated in the various cancer cell lines. In particular, the activation of negative regulators of the cell cycle (e.g., retinoblastoma 1 and retinoblastoma-like 1 and 2, etc.), and the down-regulation of genes involved in cell cycle progression (e.g., different types of cyclins) have been reported (40). These gene variations may explain the arrest of cell proliferation, which results in cell death by apoptosis or necrosis, observed in the different cancer cell lines after MSC-EV treatment (40).

To define the effect of EVs on tumor growth *in vivo*, HepG2, KS, and Skov-3 cells were subcutaneously injected into SCID mice. After tumors were established (15 mm^3^ in volume), treatment with EVs began, by means of weekly intra-tumor injections of MSC-EVs. Administration of MSC-EVs significantly inhibited tumor growth of all the tested cell lines (40).

The specificity of MSC-EVs was demonstrated by the absence of *in vitro* and *in vivo* anti-tumor effects of EVs that were derived from human dermal fibroblasts (40, 41).

Another recent paper described the effect of EVs derived from human cord blood Wharton’s jelly MSCs (hWJMSC-EVs) on the growth of T24 bladder tumors *in vitro* and *in vivo* (41). As shown for BM-MSCs, hWJMSC-EVs also inhibited cancer cell viability by cell cycle arrest, and by induction of apoptosis, in a dose-dependent fashion, both *in vitro* and *in vivo*. In this case, T24 cells were pre-stimulated with EVs prior to *in vivo* injection in nude mice. The anti-proliferative and pro-apoptotic effects were mediated by the down-regulation of Akt phosphorylation and the up-regulation of Caspase-3 cleavage (41).

In addition, EVs derived from human liver stem cells (HLSCs) have been demonstrated to have an anti-tumor effect. HLSCs inhibited the growth of HepG2 hepatoma, primary hepatocellular carcinoma, lymphoblastoma, and glioblastoma cells, both *in vitro* and *in vivo* (42). This study was the first to report a relevant role of miRNA, shuttled by stem cell-derived EVs, in the anti-tumor effect. Different approaches have been used to demonstrate the role of miRNAs in the anti-tumor effect of EVs. First of all, HLSC lines deprived of miRNA content by Dicer silencing were generated (42). These HLSC populations, and their derived EVs, showed a significant reduction of the anti-tumor miRNAs – miR223 (43, 44), miR31 (45, 46), miR122 (47–49), and miR214 (50–52). EVs derived from Dicer knock-down HLSCs showed a significant reduction of anti-tumor activities, both *in vitro* and *in vivo* (42). Another approach for demonstrating the involvement of these miRNAs in the anti-tumor activity of EV-HLSCs was the use of specific miRNA inhibitors against the anti-tumor miRNAs shuttled by EV-HLSC, such as miR451, miR223, miR24, miR125b, and miR31. This strategy resulted in a reduction of the pro-apoptotic *in vitro* activity of EV-HLSCs on hepatoma cells (42). Moreover, the relevance of miR31 and miR451 in the anti-tumor effect of EV-HLSCs was supported by experiments showing that the correspondent miRNA mimics induced tumor regression (42).

Extracellular vesicles derived from murine MSCs were also shown to significantly down-regulate the expression of vascular endothelial growth factor (VEGF) in breast cancer cells, causing an inhibition of angiogenesis both *in vitro* and *in vivo* (53). EVs derived from MSCs were shown to shuttle anti-angiogenic molecules. Specifically, they were particularly enriched in miR16, known to target VEGF (54). Treatment with MSC-derived EVs did not affect tumor cell proliferation and viability, but down-regulated the mRNA and protein levels of VEGF in tumor cells, in a dose-dependent manner (53). The transfer of miR16 from MSCs to cancer cells by means of EV has been indicated as the main mechanism for the anti-angiogenic effect of murine MSC-derived exosomes (53).

## Pro-Tumor Growth Effect of MSC-Derived EVs

When human gastric and colon cancer cell lines (SGC-7901 and SW480, respectively) were mixed with MSCs or MSC-derived EVs, and injected subcutaneously in nude mice, an increase of tumor incidence and growth was observed (55). This effect was attributed to an enhancement of cancer cell proliferation *in vivo*, as shown by an increase of the proliferating cell nuclear antigen (PCNA) positive cells in tumors. *In vitro*, the pro-proliferative effect on cancer cells was not observed, and there were no differences in the percentage of cells in the G0/G1, S, and G2/M phases between EV-treated and untreated cells. The authors observed a dose-dependent increment of VEGF and CXCR4 mRNA and protein in cancer cells at 48 h after incubation with exosomes. Indeed, VEGF and CXCR4 are critical for tumor growth and angiogenesis. These data suggested that EVs did not directly stimulate proliferation of cancer cells, but rather induced a pro-angiogenic program that could favor tumor engraftment and growth. This pro-angiogenic effect was confirmed *in vivo*, where an increment of tumor vascularization was observed. Moreover, the authors reported that EV treatment enhanced VEGF expression in cancer cells by activation of the ERK1/2 pathway. Inhibition of this pathway counteracted the increase of VEGF levels induced by EV treatment (55).

The same group that demonstrated the anti-tumor activity of hWJMSC-EVs in bladder cancer recently reported that the same EVs can in fact promote growth and aggressiveness of a renal carcinoma cell line (786-0), both *in vitro* and *in vivo* (56). In this context, after 48 h of incubation, EVs facilitated the cell cycle progression from G0/G1 to S phase. These data were confirmed *in vivo*, by detection of up-regulation of cyclin D1 expression, which favors the cell cycle transition from G1 to S phase. Interestingly, pre-treatment of EVs with RNase abrogated both the *in vitro* and *in vivo* effects of EVs on tumor cells, indicating the crucial involvement of the mRNAs, shuttled by EVs, in promoting proliferation in renal carcinoma cells. In addition, when renal carcinoma cells were mixed with EVs and subcutaneously injected in mice, the authors observed an up-regulation of hepatocyte growth factor (HGF) expression, at the mRNA and protein levels. hWJMSC-EVs containing HGF mRNA, with subsequent delivery of this mRNA into cancer cells, via EVs, may be one of the possible mechanisms of action. Moreover, after EV treatment, activation of the AKT and ERK1/2 pathways was observed in cancer cells, both *in vitro* and *in vivo*. To demonstrate the association of HGF induction with activation of AKT and ERK1/2, a c-Met inhibitor was added *in vitro* to block HGF/c-Met signaling. Under these conditions, the EV-induced activation of AKT and ERK1/2 was abrogated, with a consequent inhibition of cancer cell proliferation (56).

These results (Table 1) reveal that the same EVs can have opposite effects on different tumors, highlighting the necessity of comparative studies on different cell types in order to identify whether MSC-derived EVs exert a beneficial or a detrimental effect on the particular tumor.

**Table 1:** **EVs from different MSC source have opposite effect on different tumor types**.

Source of EVs	Tumor type	Effect on tumor growth	Reference
Human BM-MSCs	Hepatoma	Inhibition	40
	Kaposi’s sarcoma	Inhibition	
	Ovarian cancer	Inhibition	
	Gastric and colon cancer	Promotion	55
Murine BM-MSCs	Brest cancer	Inhibition	53
Cord blood Wharton’s jelly MSCs	Bladder cancer	Inhibition	41
	Renal cancer	Promotion	56
HLSCs	Hepatoma	Inhibition	42
	Lymphoblast	Inhibition	
	Glioblastoma	Inhibition	

## Different Approaches for Increasing the Anti-Tumor Effect of MSC-Derived EVs for Clinical Application

Given these variable results, it is evident that, before even beginning to consider MSC-derived EVs as a potential therapeutic tool, it is necessary to define the mechanisms of their anti-tumor activity and the application context. This requires the identification of molecules with potential healing properties for a given tumor, and the development of strategies for vesicle loading and specific targeting. As EVs protect nucleic acids from degrading enzymes, one possible approach is the *de novo* expression, or the increased expression, of specific components (mRNA, miRNA, and siRNA) with anti-tumor activity, by genetic modification of MSCs. Overexpression of specific miRNAs in the cells of origin leads to an augmented secretion of these miRNAs in EVs (39). Recently, the possibility of using EVs, purified by MSCs, as a vehicle to delivery anti-tumor miRNA has been tested, in a rodent model of malignant glioma (57). MSCs were transfected with a plasmid encoding for miR-146, and EVs were recovered 48 h later. Plasmid-expressed miRNA was thus packaged into EVs. These particular EVs were tested *in vivo* by direct intracranial injection in rats with gliosarcoma. A single intra-tumor injection of miR-146 EVs was shown to significantly reduce tumor size (57).

The characteristic of EVs of being able to cross the blood–brain barrier, may allow delivery of therapeutic substances to tumors of the nervous system. MSCs and brain parenchymal cells were shown to reciprocally communicate via EVs by transfer of miR-133b, ensuing modulation of neurite outgrowth (58). Specific targeting for neurons was achieved by Alvarez-Erviti et al. (59), who engineered EV-producing cells to express the protein Lamp2b fused to the neuron-specific RVG peptide, in order to obtain neuronal localization, and to deliver BACE-1 siRNA. In this way, they were able to obtain silencing of BACE-1, which is a beta secretase responsible for generation of toxic beta amyloid formation and deposition in Alzheimer’s disease (59). Several other studies have indicated the possibility of *ex vivo* manipulation of miRNA content of EVs, for therapeutic purposes (60–62).

Moreover, EVs may deliver biologically active proteins that could influence the phenotype of recipient cells. For example, MSCs have been successfully transduced with viral vectors so that they release functional interferon alpha (IFN-α), which is clinically used to treat various types of cancer (63). In this case, injection of MSC-producing IFN-α, the derived EVs with tropism for tumors, can provide an innovative therapy for cancer treatment, by direct delivery of IFN-α into tumors.

## Conclusions

In the contest of cancer, EVs derived from MSCs have been shown to mimic most of the beneficial and detrimental effects of the cells of origin. These opposing effects observed in different tumor types could depend on the different pathways involved. It is therefore critical to identify which molecules, shuttled by EVs, could interfere with these pathways, and therefore which kind of tumors may benefit from MSC-EV treatment. Another critical point is the timing of EV treatment. Studies based on pre-incubation of cancer cells with EVs have mainly provided information on their role in tumor engraftment and growth, but without providing a therapeutic strategy. The intra-tumor administration of EVs in an established tumor better demonstrates a therapeutic application for EVs. However, local administration may be complicated in patients, and studies are required to evaluate whether an intravenous injection of EVs is equally efficient. As in the case of MSCs, EVs may accumulate within tumors, and, therefore, could be exploited for drug delivery. Anti-tumor miRNAs and specific siRNAs are candidates for delivery by EVs. To envisage a therapeutic use of EVs, a scalable production of non-immunogenic EVs is necessary. MSCs are potential candidates for this, but the cell senescence with the subsequent cell culture passages need to be encompassed. A possible strategy, developed by Chen et al. (64) was based on oncogenic immortalization of human embryonic stem cell-derived MSCs. These authors showed a quantitative persistent production of EVs that did not contain the oncogene, and maintained the same properties of EVs derived from non-immortalized cells.

In conclusion, native MSC-derived EVs have been shown to possess therapeutic potential in some but not all tumors. The strategies to engineer EVs may be exploited to deliver anti-tumor molecules, by crossing physiological barriers.

## Conflict of Interest Statement

The authors declare that the research was conducted in the absence of any commercial or financial relationships that could be construed as a potential conflict of interest.
